# Effect of the Laying Order of Core Layer Materials on the Sound-Insulation Performance of High-Speed Train Carbody

**DOI:** 10.3390/ma16103862

**Published:** 2023-05-20

**Authors:** Ruiqian Wang, Dan Yao, Jie Zhang, Xinbiao Xiao, Xuesong Jin

**Affiliations:** 1 State Key Laboratory of Traction Power, Southwest Jiaotong University, Chengdu 610031, China; 2 School of Mechanical Engineering and Rail Transit, Changzhou University, Changzhou 213164, China; 3 Aviation Engineering Institute, Civil Aviation Flight University of China, Guanghan 618307, China; 4 State Key Laboratory of Polymer Materials Engineering, Polymer Research Institute, Sichuan University, Chengdu 610065, China

**Keywords:** sound insulation, sound-insulation material, sound absorption material, laying scheme, optimal design, high-speed train

## Abstract

The design of sound-insulation schemes requires the development of new materials and structures while also paying attention to their laying order. If the sound-insulation performance of the whole structure can be improved by simply changing the laying order of materials or structures, it will bring great advantages to the implementation of the scheme and cost control. This paper studies this problem. First, taking a simple sandwich composite plate as an example, a sound-insulation prediction model for composite structures was established. The influence of different material laying schemes on the overall sound-insulation characteristics was calculated and analyzed. Then, sound-insulation tests were conducted on different samples in the acoustic laboratory. The accuracy of the simulation model was verified through a comparative analysis of experimental results. Finally, based on the sound-insulation influence law of the sandwich panel core layer materials obtained from simulation analysis, the sound-insulation optimization design of the composite floor of a high-speed train was carried out. The results show that when the sound absorption material is concentrated in the middle, and the sound-insulation material is sandwiched from both sides of the laying scheme, it represents a better effect on medium-frequency sound-insulation performance. When this method is applied to the sound-insulation optimization of a high-speed train carbody, the sound-insulation performance of the middle and low-frequency band of 125–315 Hz can be improved by 1–3 dB, and the overall weighted sound reduction index can be improved by 0.9 dB without changing the type, thickness or weight of the core layer materials.

## 1. Introduction

In the noise control of high-speed trains, sound-insulation design is always very important. A high-speed train’s body is a complex multilayer composite structure, which includes the body profile, inner plate and core layer materials (all kinds of sound absorption/insulation materials and damping materials). Its acoustic properties are influenced by its mass, structure and material [1,2,3].

In order to achieve higher acoustic insulation performance, many studies were proposed. For example, from the perspective of optimizing acoustic structure, Yao et al. [4,5], taking typical cross-section mass and average sound transmission loss in the 400 to 3150 Hz frequency band as the optimization objectives, carried out lightweight sound-insulation design for the aluminum profile of the floor of a high-speed train, which improved the average sound insulation and weighted sound insulation by 0.8 dB and 1.0 dB, respectively, in the aforementioned frequency band. They also proposed a modal adaptive damping treatment optimization design method that effectively improved the acoustic and vibrational performance of the floor’s structure. Lin et al. [6] optimized the acoustic and vibrational performance of aluminum profiles from the aspects of the acoustic bridge, plate thickness, structural materials, etc., and obtained a structure with better acoustic and vibrational performance. Zhang et al. [7] used the wavenumber finite element method and the wavenumber boundary element method to study the influence of core sections of rectangular, triangular and trapezoidal trusses on sound transmission loss of aluminum profiles in detail. Jacek et al. [8] proposed the composite floor with a dry floating screed and a suspended ceiling, which achieved satisfactory results in airborne and impact sound insulation. Li et al. [9] proposed an orthogonally rib-stiffened honeycomb double sandwich structure with periodic arrays of shunted piezoelectric patches, which can improve the low-frequency sound insulation of the aircraft cabin and broaden the sound-insulation bandwidth.

There are also some studies from the perspective of new materials, such as by Hu et al. [10], who analyzed the influence of honeycomb core specifications, panel materials and glass bead modification on the sound-insulation performance of honeycomb sandwich panels by using the hot-pressing method and the four-sensor impedance tube method, providing data support for the selection of train body materials. Kim et al. [11,12] evaluated the sound-insulation performance of honeycomb composite panels and discussed the feasibility of replacing traditional corrugated steel plates with honeycomb composite panels in a car body. They also improved the sound-insulation effect by placing polyurethane foam in the aluminum cavity of a high-speed train’s body. Kaidouchi et al. [13] studied the sound-insulation performance of an in-plane honeycomb sandwich structure in aerospace applications and found that glass-fiber-reinforced polymer cores with fiber-reinforced plastic facing materials have better vibro-acoustic and sound transmission characteristics. Kang et al. [14] proposed an elastic layer with a low specific modulus in the middle of the core layer, which can significantly improve the broadband sound-insulation performance of the panel. Liu et al. [15] put forward the selection principle of aluminum profile surface damping material, which can predict the sound-insulation performance of an aluminum extrusion plate at the initial stage of design or put forward suggestions for improvement. Zhang et al. [16] designed a lightweight, low-frequency acoustic metamaterial (beam-like resonator) for low-frequency noise and vibration control based on the multiparameter optimization method, which has an obvious effect on the low-frequency noise and vibration control of a high-speed train’s floor structure.

To sum up, the existing research mainly improves the sound-insulation performance through material modification and structural design. However, the laying order of different materials and different structures also has an important effect on the sound insulation of the whole structure, which was often ignored in the previous research. In addition, the application of a new structure and new materials also involves a lot of new problems, such as the need to redesign space and weight accounting, recheck the strength and recheck the fire rating and insulation performance. This brings higher research, development and verification costs. If the sound-insulation performance of the whole structure can be improved simply by changing the placement sequence of materials or structures, it will bring great advantages to the implementation of the scheme and cost control.

Compared with previous studies, the research in this paper is different in that it can improve the sound-insulation performance by changing only the laying sequence of core layer materials without changing the type, thickness and density of core layer materials in the structure. Relatively speaking, this approach has almost no change to the space, thickness, weight and material type of the structure. It will bring great advantages to the implementation of the program and cost control and has a high engineering practical significance. In Section 2, taking a simple sandwich composite board as an example, the influence of changing the material laying order on the overall sound-insulation characteristics is simulated and analyzed. In Section 3, experiments are carried out to verify the simulation results of the previous section. In Section 4, the research results are applied to the sound-insulation optimization design of a high-speed train’s floor structure, and the optimization effect is evaluated.

## 2. Simulation Study on the Effect of Core Layer Material Laying Order on Sandwich Composite Panel Sound Insulation

**Figure 1:**
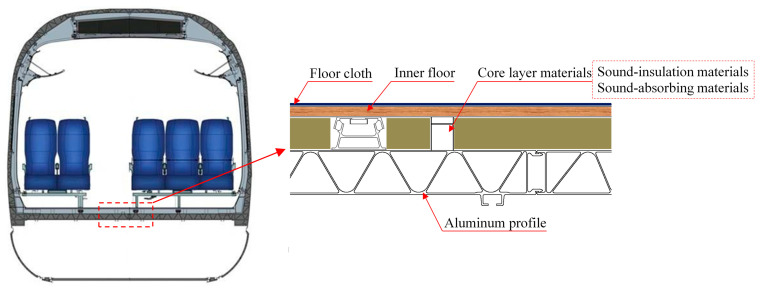
Section of a high-speed train’s floor structure.

In Figure 1, a section diagram of a high-speed train’s floor structure is provided. It can be seen that the outside of the floor structure has an aluminum profile; the inside is the inner floor; and the middle is the core layer material, which is similar to a sandwich structure on the whole. Therefore, this paper first takes a simple sandwich composite board as the object to explore the effect of the laying sequence of core layer materials on the overall sound-insulation characteristics, which can provide guidance for the subsequent sound-insulation design of high-speed train floor structures.

**Figure 2:**
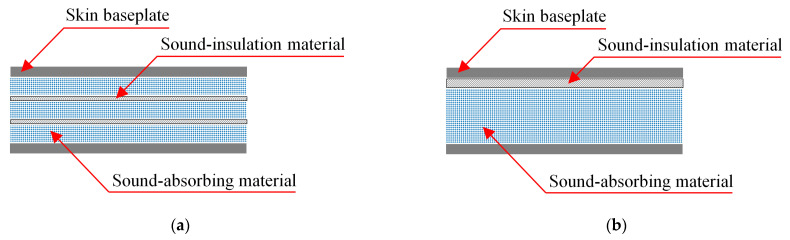

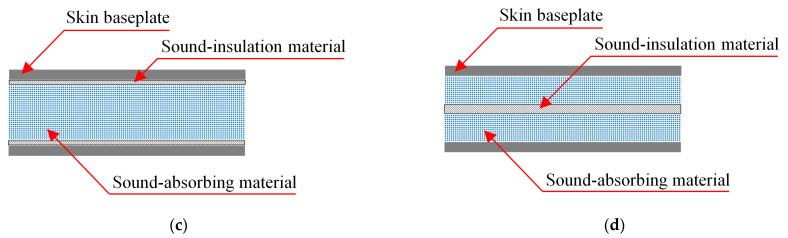
Four typical material laying schemes. (**a**) Scheme #1; (**b**) Scheme #2; (**c**) Scheme #3; (**d**) Scheme #4.

As shown in Figure 2, under the premise that the total thickness, weight and type of core layer materials remain unchanged, there are four different laying sequences: (1) sound insulation and absorption materials are uniformly stacked alternately; (2) sound-insulation materials and sound-absorbing materials are stacked separately; (3) the sound-insulation materials are concentrated in the middle, and the sound-absorbing material is distributed on both sides; (4) sound-absorbing materials are concentrated in the middle, and sound-insulation materials are distributed on both sides. In this section, corresponding comparative calculation conditions are set for these four situations to explore their influence on sound insulation.

### 2.1. Sound Insulation Prediction Model

Statistical energy analysis (SEA) is an effective method for acoustic modeling and prediction [17] that is often used in the field of sound insulation [18,19,20]. The whole system is composed of a number of statistical subsystems, and the external incentive is applied to obtain a quick response from the system. This is realized by using the commercial software VA One [21].

**Figure 3:**
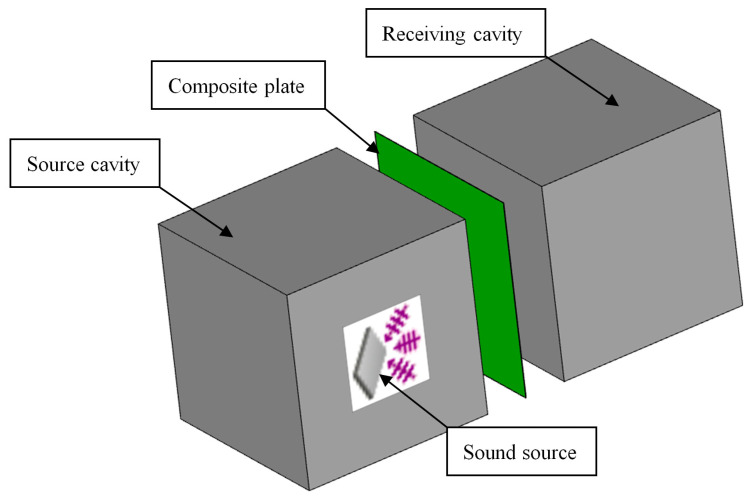
Sound insulation prediction model of sandwich composite plate.

**Figure 4:**
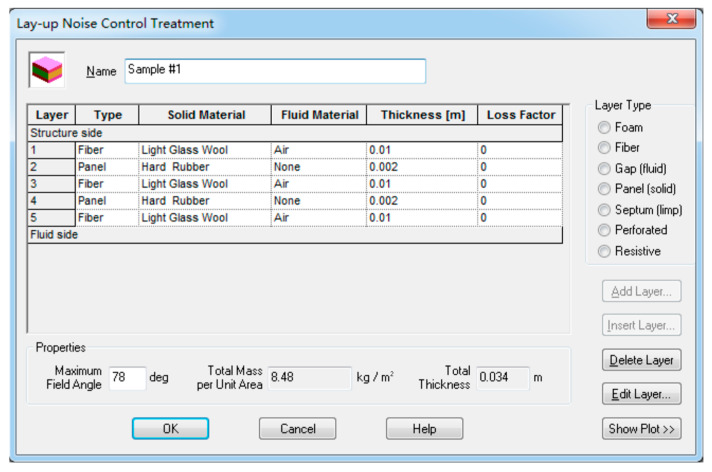
Sound package setup.

The sound cavity–sandwich plate–sound cavity insulation prediction model was established in the software VA One, as shown in Figure 3. The dimensions of both cavities are 1.0 m × 1.0 m × 1.0 m. A reverberation field is applied to one of the cavities to simulate the source excitation and to the other cavity to simulate the receiving side. The length and width of the sandwich plate are 1.0 m × 1.0 m, and it is composed of a skin substrate and core layer material (sound insulation material and sound-absorbing material), which can be realized by using the acoustic package [22,23,24] in the software, as shown in Figure 4. Model verification was carried out before starting the model.

The sound insulation of the plate structure is defined as follows:

(1) $$TL=L_{1}-L_{2}+10\log_{10}\frac{S}{A}$$

where *L*_1_ and *L*_2_ are the average sound pressure levels of the source chamber and the receiving chamber; *S* is the area of the sample; and *A* is the sound absorption coefficient of the receiving room, which can be obtained by testing the reverberation time of the receiving room according to Equation (2).

(2) $$A=\frac{0.16V_{2}}{T}$$

Here, *V*_2_ is the volume of the receiving chamber, and *T* is the reverberation time of the receiving room. The relationship between the reverberation time and the damping loss factor of the receiving chamber is as follows:

(3) $$\eta =\frac{2.2}{Tf}$$

In SEA, the difference in sound pressure level between the external and internal cavities is related to the energy density ratio of the two cavities:

(4) $$L_{1}-L_{2}=10\log_{10}\frac{E_{1}/V_{1}}{E_{2}/V_{2}}$$

By substituting Equations (2)–(4) into Equation (1), the sound insulation calculation results can be obtained, as shown in Equation (5).

(5) $$TL=10\log_{10}\left(\frac{E_{1}}{E_{2}}\frac{27.5\pi S}{\omega V_{1}\eta }\right)$$

After obtaining the calculation result of the sound-insulation frequency curve, the weighted sound reduction index *R_w_* is further calculated according to the standard [25], which is used as the single value evaluation quantity to evaluate the overall sound-insulation level of the sample.

### 2.2. Research Schemes and Results Analysis

The hard rubber and light glass wool provided in the software are used as the sound-insulation and absorption materials in the core layer, and a total of two comparison groups are set. In comparison to Group #1 and #2, a 2 mm aluminum plate and 4 mm thick plywood were used as the skin baseplate, respectively. Then, four samples with the same type of core layer materials, equal weight and thickness were set as the comparison schemes for each comparison group, and the difference was only in their laying order. Table 1 shows the basic parameters of the skin and core materials involved in the model. Table 2 and Table 3, respectively, give the concrete laying schemes of the core layer materials of the two comparison groups.

As can be seen from Table 2 and Table 3, Samples #1-1 and #2-1 are laid alternately in the core layer with sound-insulation materials and sound-absorbing materials. Sample #1-2 and #2-2 are separated soundproof materials and sound-absorbing materials; Sample #1-3 and #2-3 are soundproof materials placed in the middle of the core layer and sound-absorbing materials placed on both sides; in both Samples #1-4 and #2-4, soundproof materials are placed on both sides of the core layer, and sound-absorbing materials are concentrated in the middle of the core layer.

**Figure 5:**
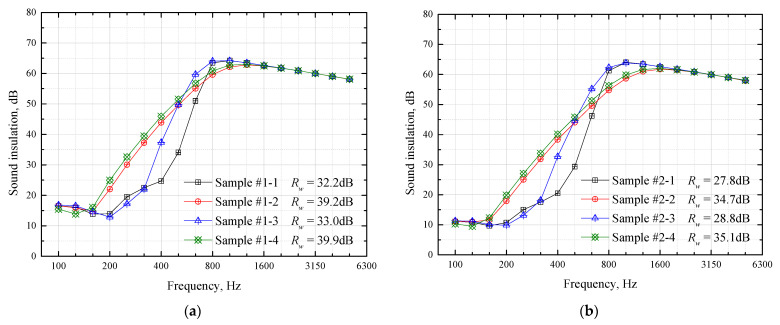
Calculation results of influence of material laying order on sandwich plate sound-insulation. (**a**) Group #1; (**b**) Group #2.

Figure 5a,b, respectively, show the sound-insulation calculation results of the comparison Groups #1 and #2. As can be seen from Figure 5a, the four samples have little difference at frequencies below 160 Hz and above 800 Hz, and the difference is mainly in the middle-frequency band between 200 Hz and 630 Hz. Among them, Sample #1-1 has the lowest intermediate frequency sound insulation; compared with Sample #1-1, the increase in Sample #1-3 is 9–13 dB in the frequency band 400–630 Hz. Compared with Sample #1-3, the increase in Sample #1-2 is 6–13 dB at 200–400 Hz, but a slight decrease occurs at 630–800 Hz. Compared with Sample #1-2, Sample #1-4 has an increase of nearly 2 dB at 200–800 Hz. From the perspective of the overall weighted sound reduction index *R_w_*, the order of the four sample values is consistent with the quality of the intermediate frequency sound-insulation level. The law in Figure 5b is basically similar to that in Figure 5a, and it is not repeated.

Furthermore, combined with the material composition of each sample, it can be found that the quality of intermediate frequency sound insulation has the greatest relationship with the concentration of sound-absorbing materials. The relationship is basically positive. Taking comparison Group #1 as an example, the total thickness of the sound-absorbing materials in 4 samples is 30 mm. Sample #1-1 is divided from 2 acoustic layers into 3 thin sound-absorbing layers with a thickness of 10 mm. Sample #1-3 is divided by a soundproof layer into 2 thin sound-absorbing layers with a thickness of 15 mm. The sound-absorbing layer of Sample #1-2 is not divided but placed separately from the sound-insulation layer without them crossing each other. The sound-absorbing layers of Sample #1-4 are also not divided but sandwiched between two soundproof layers.

**Figure 6:**
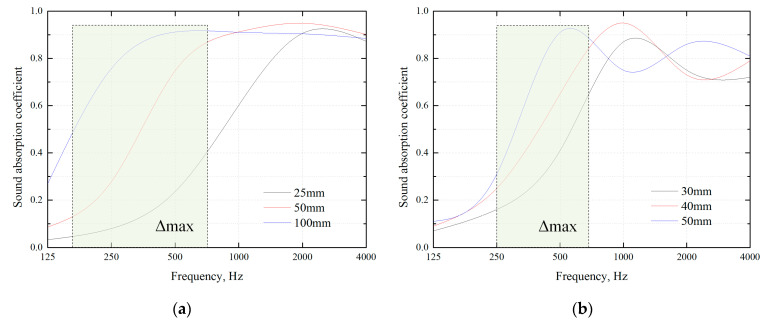
Variation in sound absorption coefficient with the thickness of the porous material. (**a**) Superfine glass wool (Volume–weight 60 kg/m^3^); (**b**) Polyurethane foam (Volume–weight 10 kg/m^3^).

The above calculation results show that for simple sandwich composite panels, the sound-insulation characteristics of the whole structure are only affected if the laying sequence is changed under the premise that the type, thickness and weight of the core layer materials are unchanged. Among them, the sound absorption material is centrally placed, significantly improving the intermediate frequency sound insulation’s performance. This is caused by the variation in sound absorption properties of the material’s thickness. As shown in Figure 6a,b, the measured results of the sound absorption coefficient with varying thicknesses of two porous materials are provided. It can be seen that with the increase in material thickness, the optimal frequency band of the sound absorption coefficient moves in the low-frequency direction, and the frequency band with the largest increase in the sound absorption coefficient happens to be located in the middle-frequency band of the sound absorption curve. In addition, the sound-insulation material is divided and evenly placed on both sides of the sound absorbing material, close to the skin substrate, which can further improve the level of sound insulation, but the improvement is not large.

## 3. Test Verification

**Figure 7:**
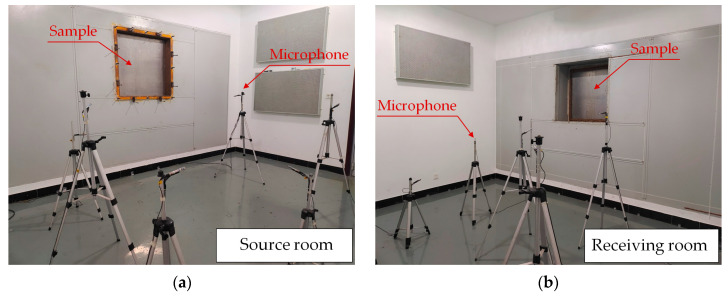
Sound-insulation test site. (**a**) Source room; (**b**) Receiving room.

Based on the double reverberation chamber method [26], the simulation calculation results in Section 2 were tested and verified. As shown in Figure 7, the test sample size was 1 m^2^, which was installed in the sound-insulation hole, and the surrounding area was sealed well with oil sludge. The sound source room and the receiving room were each equipped with 6 microphones. Before the test, a technical inspection of the number and positions of loudspeakers and microphones in the source room was completed. During the test, a diffused sound field of more than 100 dB was applied in the source room by using an undirected speaker, and the average sound pressure levels *L*_1_ and *L*_2_ of the source room and the receiving room were measured. The test frequency ranged from 100 Hz to 5000 Hz.

**Figure 8:**
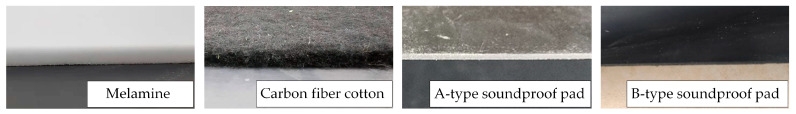
Cross-sections of the basic materials.

Two test comparison groups, #3 and #4, were set up, and two samples were set up in each comparison group, as shown in Table 4. Figure 8 shows the section of the basic sound-absorbing materials and sound-insulation materials involved. Melamine and carbon fiber cotton are both soft and porous materials that are often used to fill the core layer of train body structure and can play the role of thermal insulation and sound absorption. Soundproof pads are often used to increase the sound-insulation level for composite structures. The two types of sound-insulation pads used in this paper have different thicknesses and hardness.

**Figure 9:**
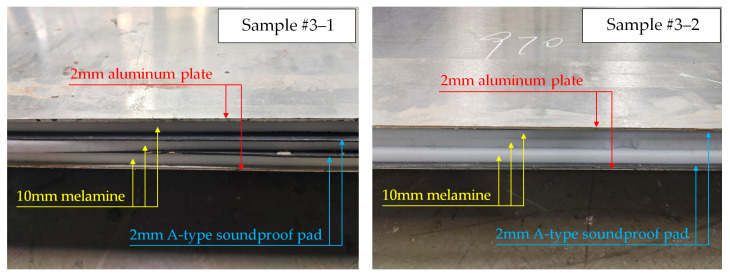

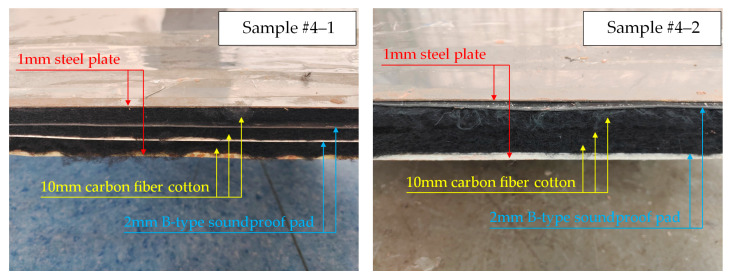
Cross-sections of the test samples.

The cross-sections of the samples are shown in Figure 9. In terms of materials, the two comparison groups had different skin baseplates, sound-insulation materials and sound-absorbing materials. Among them, Group #3 was made with a 2 mm aluminum plate as the skin baseplate and an A-type soundproof pad and melamine as sound-insulation and sound-absorbing materials in the core layer. In Group #4, a 1 mm steel plate was used as the skin baseplate, and a B-type soundproof pad and carbon fiber cotton were used as the sound-insulation and sound-absorbing materials in the core layer. In terms of the laying sequence of core layer materials, Sample #3-1 and Sample #4-1 were laid alternately with sound-insulation materials and sound-absorbing materials. However, Sample #3-2 and Sample #4-2 both concentrated sound-absorbing materials in the middle of the core layer and sound-insulation materials on both sides of the core layer.

**Figure 10:**
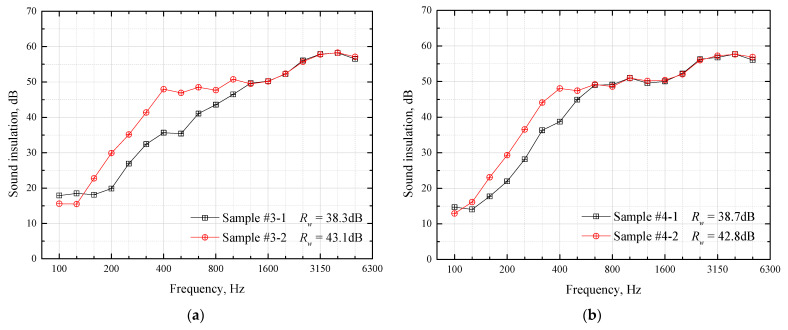
Experimental results of Group #3 and #4. (**a**) Group #3; (**b**) Group #4.

Figure 10 shows the test results of comparison Groups #3 and #4. As can be seen from Figure 10a, compared with Sample #3-1, the sound insulation of Sample #3-2 is significantly improved at 160–1000 Hz, and the improvement amount even reaches 12 dB in the frequency band 400–500 Hz. Overall, the weighted sound reduction index *R_w_* increased by 4.8 dB. The law shown in Figure 10b is similar to that in Figure 10a, so it is not repeated.

The experimental results of the simple sandwich composite plate proved that the simulation results of the previous section are correct. This indicates that, compared with “sound insulation materials and sound absorbing materials alternately placed”, the material laying scheme of “sound absorbing materials concentrated in the middle and sound insulation materials placed on both sides” is more conducive to the sound-insulation performance’s improvement in the middle-frequency band and is very beneficial to the overall weighted sound reduction index’s improvement.

## 4. Optimization Design of Floor Sound Insulation for High-Speed Train

With reference to the research conclusion of the simple sandwich composite plate mentioned above, the floor structure of a high-speed train, shown in Figure 1, was taken as an example for carrying out the sound-insulation optimization design.

**Figure 11:**
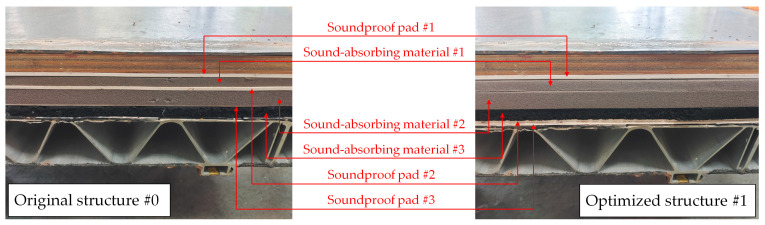
Cross-section of each floor structure.

As shown in Table 5, the first column provides the core layer material composition of the original Structure #0 of the high-speed train’s floor. It can be seen that in the original structure, sound-insulation materials and sound absorbing materials are basically in alternate laying form; then, the second column follows the principle of “sound-absorbing materials are placed centrally in the middle and sound-isolating materials are placed on both sides”, and the laying sequence of the core layer materials is adjusted to form sound-insulation optimization Structure #1. The cross-section of each structure is shown in Figure 11.

**Figure 12:**
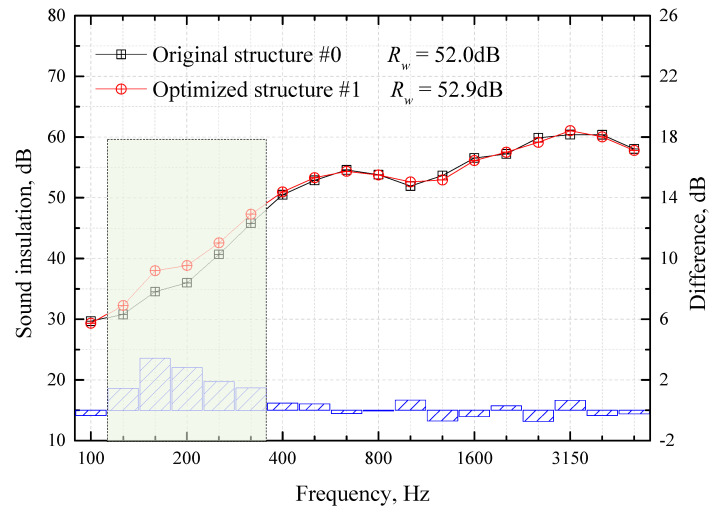
Sound-insulation optimization results for a high-speed train’s floor structure.

Figure 12 shows the sound-insulation test results of each structure in Table 5. As can be seen from the figure, compared with the original floor Structure #0, the type, thickness and weight of the core layer materials of the floor optimization Structure #1 remain unchanged. However, the change in the materials’ laying order causes the middle and low-frequency of 125–315 Hz to increase by 1–3 dB and the overall weighted sound reduction index Rw to increase by 0.9 dB.

According to the mass law, 9.9 kg of weight is needed to increase the sound insulation of this floor structure (total weight 90.6 kg) by 0.9 dB. However, by adjusting the laying sequence of the core materials, a 0.9 dB improvement in sound insulation can be achieved without adding weight, which is the main contribution of this study.

## 5. Conclusions

In this study, traditional insulation and sound-absorbing materials in a sandwich composite structure were taken as the object to study the influence of different material laying orders on the overall sound-insulation characteristics, and a high-speed train’s body structure was taken as an example to implement the sound-insulation design. The results are summarized as follows:

1. For a simple sandwich composite structure, the core layer material laying strategy of “laying sound-absorbing materials in the middle and sound-insulation materials on both sides” has a better effect on sound insulation for low and medium frequencies, and the increase is even more than 10 dB around 400 Hz, compared with “alternating laying of sound-absorbing materials and sound-insulation materials”. Furthermore, for the overall weighted sound reduction index *R_w_*, it can be increased by more than 4 dB.
2. The core layer material laying strategy of “laying sound-absorbing materials in the middle and sound-insulation materials on both sides” is applied to the sound-insulation design of the high-speed train carbody. Under the premise of not changing the type, thickness and weight of materials, the low and medium frequency sound insulations of 125–315 Hz are improved by 1–3 dB, and the overall weighted sound reduction index *R_w_* is improved by 0.9 dB.
3. By changing the laying sequence of the core layer materials, the sound-insulation performance of the composite structure can be improved without any change to the space, thickness, weight and material type of the structure. It is easy to implement, is low cost and has high engineering practical value.

## Figures and Tables

**Table 1:** Basic parameters of materials in the model.

Parameters	Aluminum Plate	Plywood	Hard Rubber	Light Glass Wool
Density (kg/m^3^)	2700	700	1100	16
Elasticity modulus (GPa)	71.0	6.0	2.3	\
Poisson’s ratio	0.33	0.25	0.49	\
Porosity	\	\	\	0.99
Flow resistivity (N.s/m^4^)	\	\	\	9000

**Table 2:** Material composition of each research scheme in Group #1.

No.	Sample #1-1	Sample #1-2	Sample #1-3	Sample #1-4
Core layer material composition	10 mm light glass wool	4 mm hard rubber	15 mm light glass wool	2 mm hard rubber
	2 mm hard rubber			30 mm light glass wool
	10 mm light glass wool	30 mm light glass wool	4 mm hard rubber	
	2 mm hard rubber		15 mm light glass wool	
	10 mm light glass wool			2 mm hard rubber

**Table 3:** Material composition of each research scheme in Group #2.

No.	Sample #2-1	Sample #2-2	Sample #2-3	Sample #2-4
Core layer material composition	20 mm light glass wool	2 mm hard rubber	30 mm light glass wool	1 mm hard rubber
	1 mm hard rubber			60 mm light glass wool
	20 mm light glass wool	60 mm light glass wool	2 mm hard rubber	
	1 mm hard rubber		30 mm light glass wool	
	20 mm light glass wool			1 mm hard rubber

**Table 4:** Material composition of each scheme for Groups #3 and #4.

No.	Group #3		Group #4	
	Sample #3-1	Sample #3-2	Sample #4-1	Sample #4-2
Core layer material composition	10 mm melamine	2 mm A-type soundproof pad	10 mm carbon fiber cotton	2 mm B-type soundproof pad
	2 mm A-type soundproof pad	10 mm melamine	1 mm B-type soundproof pad	10 mm carbon fiber cotton
	10 mm melamine	10 mm melamine	10 mm carbon fiber cotton	10 mm carbon fiber cotton
	2 mm A-type soundproof pad	10 mm melamine	1 mm B-type soundproof pad	10 mm carbon fiber cotton
	10 mm melamine	2 mm A-type soundproof pad	10 mm carbon fiber cotton	2 mm B-type soundproof pad

**Table 5:** Material composition of the original and optimized floor structure of a high-speed train.

No.	Original Structure #0	Optimized Structure #1
Core layer material composition	5 mm soundproof pad #1	5 mm soundproof pad #1
	10 mm sound-absorbing material #1	10 mm sound-absorbing material #1
	3 mm soundproof pad #2	20 mm sound-absorbing material #2
	20 mm sound-absorbing material #2	15 mm sound-absorbing material #3
	2 mm soundproof pad #3	3 mm soundproof pad #2
	15 mm sound-absorbing material #3	2 mm soundproof pad #3
Weight (kg)	24.6	24.6
Thickness (mm)	55.0	55.0

## Data Availability

The data used to support the findings of this study are available from the corresponding author upon request.

## References

[1] Song S., Lin P., Zhao Y.-J., Chen H.-W. Prediction of sound transmission loss of composite floor structures of high speed trains. Proceedings of the INTER-NOISE 2017-46th International Congress and Exposition on Noise Control Engineering: Taming Noise and Moving Quiet.

[2] Deng T.-S., Sheng X.-Z., Jeong H., Thompson D.J. (2021). A two-and-half dimensional finite element/boundary element model for predicting the vibro-acoustic behaviour of panels with poro-elastic media. J. Sound Vib..

[3] Zhang J., Yao D., Wang R.-Q., Xiao X.-B. (2021). Vibro-acoustic modelling of high-speed train composite floor and contribution analysis of its constituent materials. Compos. Struct..

[4] Yao D., Zhang J., Wang R.-Q., Xiao X.-B., Guo J.-Q. (2019). Lightweight design and sound insulation characteristic optimisation of railway floating floor structures. Appl. Acoust..

[5] Yao D., Zhang J., Wang R.-Q., Xiao X.-B. (2020). Vibroacoustic damping optimisation of high-speed train floor panels in low- and mid-frequency range. Appl. Acoust..

[6] Lin L.-Z., Ding Z.-Y., Zeng J.-K., Zhang C.-X. (2016). Research on the transmission loss of the floor aluminum profile for the high-speed train based on FE-SEA hybrid method. J. Vibroeng..

[7] Zhang Y.-M., Thompson D., Squicciarini G., Ryue J., Xiao X.-B., Wen Z.-F. (2018). Sound transmission loss properties of truss core extruded panels. Appl. Acoust..

[8] Nurzyński J., Nowotny Ł. (2023). Acoustic performance of floors made of composite panels. Materials.

[9] Li S., Xu D., Wu X., Jiang R., Shi G., Zhang Z. (2022). Sound insulation performance of composite double sandwich panels with periodic arrays of shunted piezoelectric patches. Materials.

[10] Hu Q.-L., Bian G.-F., Qiu Y.-P., Wei Y., Xu Z.-Z. (2001). Sound insulation properties of honeycomb sandwich structure composite for high-speed train floors. J. Text. Res..

[11] Seockhyun K., Taegun S. (2010). Sound insulation performance of honeycomb composite panel for a tilting train. Trans. Korean Soc. Mech. Eng. A.

[12] Kim S.H., Seo T.G., Kim J.T., Song D.H. (2011). Sound-insulation design of aluminum extruded panel in next-generation high-speed train. Trans. Korean Soc. Mech. Eng. A.

[13] Kaidouchi H., Kebdani S., Slimane S.A. (2022). Vibro-acoustic analysis of the sound transmission through aerospace composite structures. Mech. Adv. Mater. Struct..

[14] Kang L., Sun C., An F., Liu B. (2022). A bending stiffness criterion for sandwich panels with high sound insulation and its realization through low specific modulus layers. J. Sound Vib..

[15] Liu X.-B., Yang Y., Le V. (2014). Airborne sound insulation of aluminum extrusion structural walls of an urban rail train. Noise Control Eng. J..

[16] Zhang J., Yao D., Wang P., Wang R.-Q., Li J., Guo S.-Y. (2022). Optimal design of lightweight acoustic metamaterials for low-frequency noise and vibration control of high-speed train composite floor. Appl. Acoust..

[17] Lyon R.H., DeJong R.G., Heckl M. (1995). Theory and Application of Statistical Energy Analysis.

[18] Oliazadeh P., Farshidianfar A., Crocker M.J. (2022). Experimental study and analytical modeling of sound transmission through honeycomb sandwich panels using SEA method. Compos. Struct..

[19] Huang X.-F., Lu Y.-M., Qu C., Zhu C.-H. (2021). Study on Sound Transmission across a Floating Floor in a Residential Building by Using SEA. Arch. Acoust. J. Pol. Acad. Sci..

[20] Hyoseon J., Carl H. (2018). Prediction of sound transmission in long spaces using ray tracing and experimental Statistical Energy Analysis. Appl. Acoust..

[21] (2012). VA One 2012 User’s Guide.

[22] Su J.-T., Zheng L., Lou J.-P. (2020). Simulation and Optimization of Acoustic Package of Dash Panel Based on SEA. Shock Vib..

[23] Hossein S., Abolfazl K., Amin M. (2022). A practical procedure for vehicle sound package design using statistical energy analysis. Proc. Inst. Mech. Eng. Part D J. Automob. Eng..

[24] Zhang X.-X., Wu X.-R., Cheng Y.-H., Jin H.-Y., Zhang J. (2012). Application Research of Statistical Energy Analysis on Vehicle Sound Package. FISITA World Automotive Congress.

[25] (2013). Acoustics—Rating of Sound Insulation in Buildings and of Building Elements—Part 1: Airborne Sound Insulation.

[26] (2021). Acoustics—Laboratory Measurement of Sound Insulation of Building Elements—Part 2: Measurement of Airborne Sound Insulation.

